# Clinotator: analyzing ClinVar variation reports to prioritize reclassification efforts

**DOI:** 10.12688/f1000research.14470.2

**Published:** 2018-06-20

**Authors:** Robert R. Butler III, Pablo V. Gejman

**Affiliations:** 1 Genomic Health Initiative, NorthShore University HealthSystem, Evanston, Illinois, 60201, USA; 2 Department of Psychiatry and Behavioral Neuroscience, Pritzker School of Medicine, University of Chicago, Chicago, Illinois, 60637, USA

**Keywords:** ClinVar, variation, clinical variant, pathogenic, benign, variant interpretation, variant reclassification, pathogenicity

## Abstract

While ClinVar has become an indispensable resource for clinical variant interpretation, its sophisticated structure provides it with a daunting learning curve. Often the sheer depth of types of information provided can make it difficult to analyze variant information with high throughput. Clinotator is a fast and lightweight tool to extract important aspects of criteria-based clinical assertions; it uses that information to generate several metrics to assess the strength and consistency of the evidence supporting the variant clinical significance. Clinical assertions are weighted by significance type, age of submission and submitter expertise category to filter outdated or incomplete assertions that otherwise confound interpretation. This can be accomplished in batches: either lists of Variation IDs or dbSNP rsIDs, or with vcf files that are additionally annotated. Using sample sets ranging from 15,000–50,000 variants, we slice out problem variants in minutes without extensive computational effort (using only a personal computer) and corroborate recently reported trends of discordance hiding amongst the curated masses. With the rapidly growing body of variant evidence, most submitters and researchers have limited resources to devote to variant curation. Clinotator provides efficient, systematic prioritization of discordant variants in need of reclassification. The hope is that this tool can inform ClinVar curation and encourage submitters to keep their clinical assertions current by focusing their efforts. Additionally, researchers can utilize new metrics to analyze variants of interest in pursuit of new insights into pathogenicity.

## Introduction

The dbSNP database ^1^ currently contains over 300 million reference SNPs, and dbVar ^2^ adds over 5 million variant regions to the documented plasticity of the human genome. ClinVar ^3^ is small by comparison, documenting the clinical impact of 400,000 variants. This may seem like a far simpler task; however, the substantial impact of these clinical variants on the lives of patients places a heavier burden on the level of evidence gathering required. Add to this the fragmented nature of the evidence—spread out across publications, databases, predictive software analysis and in individual health records—meaning each of these ClinVar records becomes its own meta-data analysis ^4^. ClinVar, ClinGen ^5^, and the American College of Medical Genetics and Genomics (ACMG)/Association for Molecular Pathology (AMP) ^4^ have done an excellent job formulating assertion criteria that allows for a comprehensive analysis of all available data, collating them into a standardized classification. While this became the minimum standard upon its inception, there is still a backlog of older assertions with ill-defined criteria or those missing a specification altogether. Many of these would benefit from submitter reclassification based on the more recent standards.

Given the inconsistent amounts of variant data across the genome and the rapid generation of new studies, the significance of variants also changes at an accelerated pace ^6^. Put in statistical terms, the ClinVar clinical significance represents an estimate of the true population significance, and current estimates are based on limited, often private datasets. Clinical assertions based on insufficient evidence can persist in public databases and consequently seed misinformation into future interpretations ^7, 8^. Recently, in the field of cardiovascular disease, there have been several high-profile instances of cardiovascular variants deemed to be highly pathogenic, yet not segregating with disease ^7, 9, 10^. This unfortunate outcome is inevitable owing to the aforementioned reasons and illustrates a key issue: the continual need to share and reconcile new information with old data and reclassify clinical assertions on a regular basis ^6, 11^. Several initiatives ^5, 12– 16^ have had success in encouraging the public sharing of datasets and new studies. In many of the instances of assertion discordance, consensus has been achieved simply by sharing evidence previously unavailable to one party ^7, 11, 17^. Harrison *et al.* found that 87.2% of discordant variants were resolved by reassessment and data sharing ^11^. New public data has recently been leveraged with private datasets to identify misclassified variants on the basis of variant penetrance given disease prevalence ^18^. However the majority of these reclassification efforts still rely on access to private data, which will continue to be an unavailable to most researchers for the foreseeable future.

As a clinician or researcher looking to utilize ClinVar, its depth and sophistication present a daunting learning curve. This is necessary, as ClinVar houses not only assertions, but evidence, literature, and an impressive amount of cross-reference material ^3^. As Yang *et al.* ^19^ have suggested, this makes the process of evidence interpretation challenging on an individual variant level and the batch processing of variants even more so. ClinVar itself has provided a utile web interface and simplified data structures for programmatic use ^20^. To the same end, other tools have been developed to address both aims: to easily browse variations and compare curations ^21, 22^, or import and manipulate flattened ClinVar data for variant analysis ^23, 24^. While the browsing tools allow for user-friendly and web-hosted comparison, they do not provide the throughput to analyze large datasets. Conversely, the local database tools allow for deep analysis on large variant sets, but require a significant amount of programming experience and local computational resources to access and operate.

Clinotator is unique in that it provides largescale batch analysis without necessitating a large local computational resource or deep programming knowledge. It can quickly generate simple annotation tables, annotate vcf files, or be integrated into annotation pipelines with little overhead. The goals were two-fold: (i) deliver filtered ClinVar information for each variant, focusing on clinical assertions being made about the variant; and (ii) generate several metrics by which the robustness and consistency of the evidence can be gauged for the overall clinical assertion. Clinotator’s quantification of assertion evidence takes into account significance type, submission age and submitter expertise category for a standardized scoring of clinical impact based on the five ACMG/AMP descriptors of Mendelian disorders: Benign (B), Likely Benign (LB), Uncertain Significance (US), Likely Pathogenic (LP) and Pathogenic (P) ^4^.

Our aim is for Clinotator to be useful in a number of capacities, including prioritizing variants that need reclassification, guiding submitter reconciliation efforts or simply identifying discordant variants for future research targets. Since it is based entirely on data available in ClinVar, it requires no private dataset or access to external resources. To demonstrate its utility, we examined test sets of two-star, three-star, and four-star variants (per ClinVar’s review status star ratings) and variants in ClinVar with “Conflicting Interpretations of pathogenicity” (CI). Clinotator was able to confirm recently published concordance trends ^6, 11, 19^, and identify several groups of discordant variants for further investigation. It accomplished this efficiently, using a large-scale systematic approach with a minimal computational effort.

## Methods

### Implementation

***Metric calculation.*** Clinotator collects a variety of characteristics from ClinVar and generates four additional metrics ( Table 1). The core component of these metrics is the Clinotator raw score (CTRS), generated as the sum of a variant’s weighted individual clinical assertions ( *i*):

$$CTRS=\sum_{i=2}^{n}x_{i}d_{i}s_{i}$$

$$x_{i}=\{\begin{array}{lll} \begin{array}{l} B=-6\\ LB=-3\\ US=-0.3\\ LP=3\\ P=6 \end{array} & |\;\;d_{i}=\{\begin{array}{l} 1;n<2\\ \frac{11-n}{10};2\leq n\leq 6\\ 0.5;n>6 \end{array} & |\;\;s_{i}=\{\begin{array}{l} 1.25\;;Practice\;Guideline\\ 1.10\;;\;Reviwed\;by\;Expert\;Panel\\ 1.0\;;\;Criteria\;Provided\\ 0\;;\;Otherwise\;(No\;Assertion) \end{array} \end{array}$$

**Table 1.** Metrics provided by Clinotator software.

Metric Name	Symbol	Description
*ClinVar Metrics*		
ClinVar Variation ID	VID	Identifier supplied by ClinVar for each variant entry.
dbSNP reference SNP ID	rsID	Identifier supplied by dbSNP for each reference SNP record. Returned from a successful VID lookup.
ClinVar Clinical Significance	CVCS	Clinical significance reported by ClinVar. Ratings metrics are based on the five ACMG/AMP recommended classifications for Mendelian disorders.
ClinVar Stars	CVSZ	Star rating given by ClinVar. Ranges from zero to four.
ClinVar Number of Clinical Assertions	CVNA	Number of ClinVar Submissions possessing a clinical assertion (with criteria provided).
ClinVar Conditions/ Diseases	CVDS	Conditions reported to be associated with this variant.
ClinVar Alternate Allele	CVAL	Alternate allele connected with ClinVar variation report.
ClinVar Last Evaluated	CVLE	Date the clinical significance of the variation report was last evaluated.
ClinVar Variant Type	CVVT	Type of variation in ClinVar. Currently defined as either "Simple" with a single AlleleID or "Haplotype" if multiple AlleleIDs.
*Clinotator Metrics*		
Clinotator Raw Score	CTRS	A weighted metric of pathogenicity based on submitter type, assertion type and assertion age.
Average Clinical Assertion Age	CTAA	Clinical assertions with criteria provided are counted, and their average age is calculated.
Clinotator Predicted Significance	CTPS	A predicted clinical significance based on the weighted distribution of all two-star variants in ClinVar with two or more clinical assertions.
Clinotator Reclassification Recommendation	CTRR	Ranked reclassification priority based on the difference between the CVCS and the CTPS. Scores range from zero to three in escalating priority.
vcf_match	-	A special field for identification of multiple alleleIDs in a haplotype variation report. This is not included in vcf annotations.

The assertion weight factor ( *x _i_*) was chosen such that a certain multiple of the next lowest priority significance would be less than or equal to the value of the current significance. Initial values of US were tried from -0.5 to -0.1 in increments of 0.1, ultimately defined at -0.3. The assertion weight factor for LB was tried as several multiples of US (4, 5, 6, 10, 12, and 20). A 10-fold multiple, with LB equal to -3, eliminated overlap between US and LB distributions. The value of B was tested at a range of multiples of LB (3/2, 5/3, 2, 3, and 4), and fixed at a 2-fold value, with a B assertion weight of -6. No multiple of US could attain LP, which was set as the equivalent positive value to LB; and P was set 2-fold higher than LP (after trying a range of multiples as with B). Further rationale of weights is continued in the Results and Discussion sections. The age of the assertion factor ( *d _i_*) reduces the assertion weight over time after a buffer. For the first 2 years, there is no penalty, then there is a 10% reduction gradation in weight per year through 6 years, at which point the penalty stays at a static 50% reduction thereafter. The submitter class factor ( *s _i_*) is weighted based on ClinVar submitter category as curated by ClinGen, with regular clinical assertions by genetic testing laboratories unweighted at 1.00, expert reviewers receiving a 1.10 and practice guidelines receiving a score of 1.25.

Note that the CTRS metric only includes clinical assertions where the submitter has published a defined assertion criteria on the ClinVar website. Literature-only submissions such as those from OMIM are filtered out as they are a type of evidence, and not a clinical assertion. Assertions made without assertion criteria or with incomplete data are also omitted, as the reliability of these assertions is unknown.

The Clinotator average assertion age (CTAA) is the mean age (in years) of valid clinical assertions. Each assertion’s age is calculated at the time of Clinotator script execution as the number of years since the clinical significance last evaluation date. Assertions without a last evaluation date are omitted.

The Clinotator predicted significance (CTPS) is a predicted clinical significance based on the CTRS scores of variants in ClinVar with two or more valid clinical assertions. A dataset of all variants that score two stars in ClinVar and have a Mendelian significance was used as a calibration for the category ranges. For the purposes of this calibration, variants with a Pathogenic/Likely pathogenic (PLP) or Benign/Likely benign (BLB) overall significance were excluded as they could not definitively be placed in either category. Additionally, two-star variants with fewer than two clinical assertions with assertion criteria were excluded. Using this filtered calibration dataset, the bounded regions for each CTPS category were set based on a combination of ClinVar star criteria and non-parametric prediction intervals (PI). The lower bound of each range was set at 2[(assertion weight) * (0.7) * (1.0)]; namely, the minimum ClinVar qualification for two stars with both assertions being no more than 5 years old. The quantiles of each distribution as well as the PIs were examined for a range of confidences. The PI for each clinical significance was chosen as the highest possible confidence that aligned to the above established lower bound. Calculations were conducted in R ^25^, and the non-parametric PIs were defined as the *c* ^th^ and *r* ^th^ values in each distribution, where ^26, 27^:

$$c=trunc\left(\frac{\alpha }{2}\times \left(n+1\right)\right)\;|\;r=n-c+1$$

The Clinotator reclassification recommendation (CTRR) is a ranked reclassification priority based on the absolute difference between the ClinVar clinical significance (CVCS) and the CTPS. This field uses the seven values of clinical significance associated with Mendelian diseases (B, BLB, LB, US/CI, LP, PLP, P), valued one through seven. For the purposes of reclassification, CI is scored the same as US. Each shift along the scale increases the rank by one, and a transition between overall zones (all benign ⇔ US/CI ⇔ all pathogenic) adds an additional point.

$$CTRR=\{\begin{array}{l} \;.\;\text{-}\;\text{Insufficient information for a recommendation}\\ \text{0}\;\text{-}\;\text{Reclassification}\;\text{unlikely, consistent identity}\\ \text{1}\;\text{-}\;\text{Low priority reclassification, minor change without clinical impact}\\ \text{2}\;\text{-}\;\text{Medium priority reclassification, minor change of some clinical impact}\\ \text{3}\;\text{-}\;\text{High}\;\text{priority}\;\text{reclassification, significant}\;\text{change}\;\text{in}\;\text{clinical}\;\text{impact} \end{array}$$

The total number of points is capped at three. Rankings range from zero to three, in escalating degree of inconsistency. A CTRR is only calculated with at least two valid clinical assertions with criteria.

***Software structure.*** The functional components of Clinotator are contained in four modules and a global variables file. The main program, *clinotator.py*, handles the I/O, errors and options for various file types.

The *getncbi.py* module handles querying of the E-utilities database servers ^28^. It splits the input list into batches if necessary (default eLink batch size of 1000) and posts to the Entrez history server. It then fetches xml records in batches (default eFetch batch size of 4500). It handles some minor connection interruptions and gives three retries per batch before giving up. Returned batches are added to a list of xml objects to be handled by *variation.py*.

The *variation.py* module defines the VariationClass object, and its methods parse ClinVar xml records and calculate the scoring metrics, which are then stored as instance variables. *clinotator.py* then utilizes pandas to collect and organize tabled data for output. As the ClinVar xml format is highly sophisticated, it does not frequently lend itself to flattening without considerable database structure. The construction of *variation.py* will allow for future modification, and storage of additional ClinVar xml data as class attributes, allowing for significant backend manipulation with a minimal footprint on the local machine.

The *vcf.py* module is dedicated to the handling of vcf as an input type. It stores the header and adds the new INFO field definitions for the annotation in the output file. The rsIDs in the ID column of the vcf are then sent through the rsID input method. After the annotation table has been created in *clinotator.py*, *vcf.py* matches annotations to vcf calls by rsID and alternate allele combination. Alternate alleles are handled as lists (and ClinVar haplotypes are handled as list instance objects), so multi-allelic loci are correctly labeled with their appropriate ClinVar report. Haplotypes are identified as such, but the ‘vcf_match’ field ( Table 1) is omitted from the vcf annotation. The other 12 fields are added to the INFO field as outlined in the vcf version 4.3 standards ^29^.

The *global_vars.py* file supplies a location for most static variables in the program, including several dictionaries of calibration values. Most of these values do not need any modification, but can be; for instance, download batch sizes from NCBI. If the default values result in frequent http errors, the batch size can be reduced. The maximum eLink batch size (for rsID and vcf types) is 1000 ids, while the maximum eFetch batch size is theoretically 10,000 ids. Both are set to lower levels to reduce the incidence of http errors and can be throttled based on available bandwidth.

### Operation

Clinotator was designed in a Linux environment and implemented in Python (2.7 or ≥3.4) ^30^, and can run in similar OSX and Windows Python environments. The required modules are pandas (0.20.0 minimum, 0.22.0 recommended) ^31^ and biopython (1.70) ^32^. It can be run on a personal computer with relatively modest system requirements; a minimum of 2 GB available RAM. The command line interface requires three pieces of information: (i) the type of input file, (ii) the file itself, and (iii) your email address. The input file can be one of three types: an rsID list using dbSNP reference SNP identifiers; a Variation ID (VID) list using ClinVar identifiers; or a vcf file. In each case, multiple files can be included and will be processed in batches. If using a list type file, it should be a plain text file with a list of identifiers, one per line. The email is required by NCBI/biopython.

The preferred input file types are a VID list or a vcf file. The rsID list alone is inherently ambiguous, as multi-allelic rsIDs can have several VIDs associated, one with each allele. The rsID to VID conversion is not 1:1, so the table file generated will return rows for all possible VIDs associated with the rsID. Thus an rsID generated table may require additional matching using the alternate allele column (CVAL). However, vcf files will only be annotated with the correct rsID/alternate allele combination, preventing a mix-up for the vcf input type. Conversely some VIDs have multiple rsIDs, either because they are a haplotype variant, or due to other complications with rsID curation. The ‘vcf_match’ field addresses this reverse situation by identifying all rsIDs associated with a VID and its haplotype allele status.

Additionally, the user can specify several options. A highly recommended log option (--log) generates a text file with the warnings from the run. A more extensive long log file (--long-log) can be specified for annotation details. Both log files supersede the terminal annotation warnings that occur when Clinotator finds missing xml data in ClinVar records in the default (no log) mode. The log files are written in append mode, so batch runs or multiple runs of Clinotator in the same folder can generate a significantly large log file. Users can also specify the output file prefix (the default is “clinotator”), which will label the output “tsv”, “anno.vcf”, and “log” files.

In all cases, a tab-delimited table file will be produced. The columns will be the fields in Table 1. If a vcf file is selected, Clinotator will generate an additional annotated output vcf file. Annotations are concatenated in the INFO field from Table 1. Multi-allelic input variants will include comma-separated values specific to each minor allele. For further details about installation and usage, see the github repository for this project ( *Data and software availability section*).

### Further analysis of data

All VID lists used in analysis were generated at ClinVar, using the search filters and downloading a UI list in text format. The set of all variants with at least two stars was generated February 24 ^th^, 2018, and the set of all CI variants was generated on February 27 ^th^, 2018. Both sets of variants were analyzed with Clinotator, and split into two-star, three-star, four-star and CI sets. Additional computational analysis was done using dplyr, ggplot2, ggExtra, gridExtra, and RColorBrewer R packages ^33– 37^. The February datasets were reanalyzed during the review process, June 1 ^st^, 2018.

## Results

### Computational performance

A test set of 10,000 VIDs, was run on a system with a single core from an i7-4770 CPU with 16 GB of available memory. Clinotator averaged 1.79 min to complete, 87% of which comprised the NCBI query and download time. The greatest limitation to run time is the bandwidth of the connection to the NCBI databases. When running the list of all variants with at least two stars in ClinVar (>50,000), the run time never exceeded 15 min, with a post-download parsing time of around 90 s. As Clinotator keeps the NCBI xml results in memory, there can be a substantial memory usage. At the time of writing, the entire ClinVar xml set is approaching 6 GB. Loading the entire set into memory is doable with at least 8 GB of memory, though it is recommended that you batch your queries in this rare case. More typical usage for subsets of ClinVar or batch vcf annotations should not pose a memory issue.

Batch annotation of vcf files is similarly efficient, working on single or multi-sample vcfs. Given the set of seven multi-sample, exonic vcf files available at the 1000 Genomes project, Clinotator was able to generate a variant table and annotate output vcf files for all seven files (15,171 total rsIDs) in an average of 3.94 min, 68% of which was NCBI query and download time. A potential speed limitation to vcf-based annotation is that NCBI is queried for each input vcf file, resulting in duplicate queries of common variants, but the tradeoff is not having to create a local query storage file that may potentially become very large if hundreds or thousands of vcf files are being analyzed in a pipeline. If higher throughput is required, it may be more efficient to consider a variant database structure which can return a non-redundant list of total database rsIDs and utilize the list rsID method to generate a reference table.

### Characteristics of all ClinVar variants with at least two stars

A total of 47,854 variants were identified with two or more stars in ClinVar and at least two clinical assertions, with 23 four-star, 5,807 three-star and 42,024 two-star variants. There was no discernible trend in the mean CTNA across the two-star, three-star, CI, and the calibration variant sets: 3.1 ± 1.6, 4.0 ± 3.0, 3.6 ± 2.1, and 2.8 ± 1.2. The mean CTAA across the same groups was: 1.5 ± 0.9, 1.6 ± 0.6, 1.4 ± 0.8, and 1.4 ± 0.9. Overall, variant values of 3.3 ± 1.9 assertions per variant, and an average of 1.5 ± 0.9 years old are not significantly different in these sub-groups. This points to a general continuity in ClinVar, encouraging for previous reports of concordance between different clinical labs and expert review panels ^19^. The only exception is the outlier case of the four-star variant set, with a mean CVNA of 7.4 ± 3.0 and a mean CTAA of 3.5 ± 0.9. These variants are a particular group of well-documented *CFTR* variants, though the practice guideline assertion has not been reevaluated since 2004.

**Figure 1.**
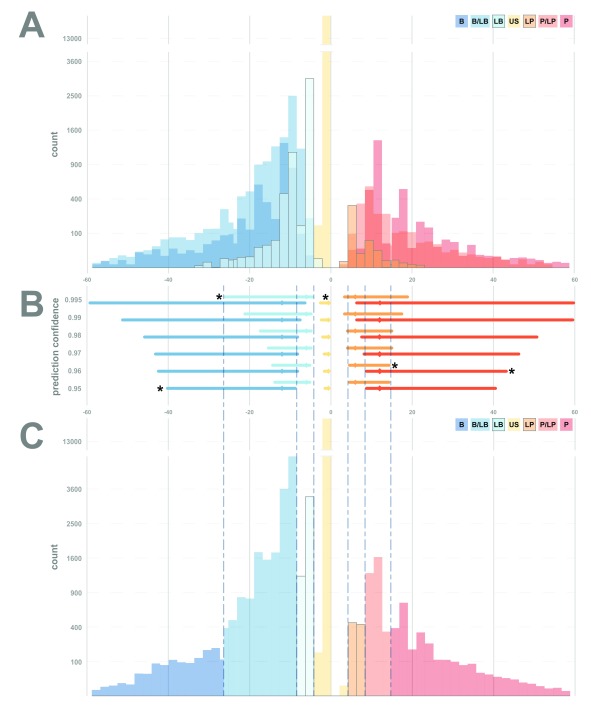
Clinotator raw score (CTRS) distribution of two-star ClinVar variants. ( **A**) All two-star variants, plotted according to CTRS, and colored based on the seven ClinVar clinical significance designations: Benign (B), Benign/Likely benign (BLB), Likely benign (LB), Uncertain Significance (US), Likely pathogenic (LP) Pathogenic/Likely pathogenic (PLP) and Pathogenic (P). ( **B**) Prediction intervals (PI) for the five primary Mendelian clinical significances (B, LB, US, LP and P). Intervals plotted by CTRS value, using five different interval confidences (vertical axis). The optimal confidence interval for each clinical significance is marked with an asterisk. ( **C**) All two-star variants plotted according to Clinotator Raw Score, and colored based on the seven Clinotator predicted significance ranges (B, BLB, LB, US, LP, PLP and P) after calibration with prediction intervals. Dashed lines denote prediction interval boundaries from ( **B**).

The two-star variants are graphed by CTRS in Figure 1A. The distributions of CTRS widely overlap and significantly skew towards overlarge outliers. The US group is the exception, with a leptokurtic distribution. Notably, despite the weighting of B and P assertion types by twice as much as their “likely” counterparts, the distributions of variants of each zone remain resolutely overlapped. The BLB distribution in particular seems both the largest and the most far ranging, extending beyond the B group. While the P group is slightly more distributed above its family members, the LP and PLP distributions, the PLP distribution still spreads over almost the entire positive side of the spectrum. As the PLP or BLB rating in ClinVar is based on a single piece of each type of evidence, there is not a quantification of how much P/B and how much LP/LB evidence is factored into each assessment.

### Clinotator calibration using control distributions

A total of 28,087 variants were two-star variants that qualified to be in the five control groups ( Table 2). These variants were used to calculate the five PIs depicted in Figure 1B. For each range, the quantiles and PIs were chosen as described above. Given the fixed lower bounds defined by two-star status in ClinVar, the confidence of every PI exceeds the similarly bounded median-centered quantile range, excepting the US category (the center 99.8% of the US distribution is larger than the 99.5% confidence PI). As the US category has no lower bound, its upper bounds are defined by the lower bounds of LB and LP categories, which are outside the entire US control distribution, resulting in a PI confidence greater than 99.99%, still not covering the full width between LB and LP. The likelihood of a US variant falling outside the chosen range is small.

**Table 2.** Control groups for Clinotator calibration. CTRR, Clinotator Reclassification Recommendation. B, Benign; LB, Likely Benign; US, Uncertain Significance; LP, Likely Pathogenic; P, Pathogenic; PI, prediction interval.

	N	Median	Quantile in Distribution	Quantiles (%)	Prediction Interval	PI confidence (%)	Percent in Tier Below	Percent in Tier Above	Percent with CTRR = 0	Percent with CTRR = 1	Percent with CTRR = 2	Percent with CTRR = 3
**B**	4755	-12.6	-37.8, -8.4	3, 97	-40.2, -8.4	95.0	-	87.6	8.9	88.7	2.4	0
**LB**	5233	-6	-24, -4.2	0.3, 99.7	-26.7, -4.2	99.5	38.5	0.0	61.4	38.2	0.3	0
**US**	14011	-0.6	-2.8, -0.4	0.1, 99.9	-2.6, -0.4	99.5	0.0	0.0	100.0	0.0	0.0	0
**LP**	502	5.7	4.2, 14.4	2, 98	4.2, 14.7	96.0	0.0	20.9	77.7	18.9	3.4	0
**P**	3586	12	8.4, 44.0	2, 98	8.4, 44.4	96.0	55.0	-	45.0	53.3	1.7	0

The resulting CTPS intervals are shown in Figure 1C, with the BLB and PLP intervals defined by the overlapping PIs. It is worth noting that the overlap between B and LB is much wider than that between P and LP, which reflects the greater overlap of control B and LB distributions. This overlap disparity is observable for all fixed PI confidences individually and in the mixed-confidence PI model used in the final calibration ( Figure 1B). Defining BLB and PLP groups with this approach has the advantage of classifying the BLB and PLP quantitatively in a range that cannot be called as either classification by the given confidence, with both classifications exceeding 95% confidence. For this purpose, the overlapping regions of PLP and BLB exist—not as yet another classification bin—as a measure of plasticity of borderline assertions. The quantitative nature of the CTRS also allows a given variant to transition out of the overlap should enough additional assertions arise, or if sparse limited assertions are not updated.

A potential concern for non-parametric PIs is that they are inaccurate for values outside the control distribution. However, as the lower bounds are not defined by the PI, only the upper bounds are vulnerable to extreme outliers. Regardless of how far outside the upper interval boundary a given variant may fall, the CTPS determination remains the same, limiting the potential for outliers to impact the reclassification score.

### Clinotator reclassification and ClinVar two-star variants

**Figure 2.**
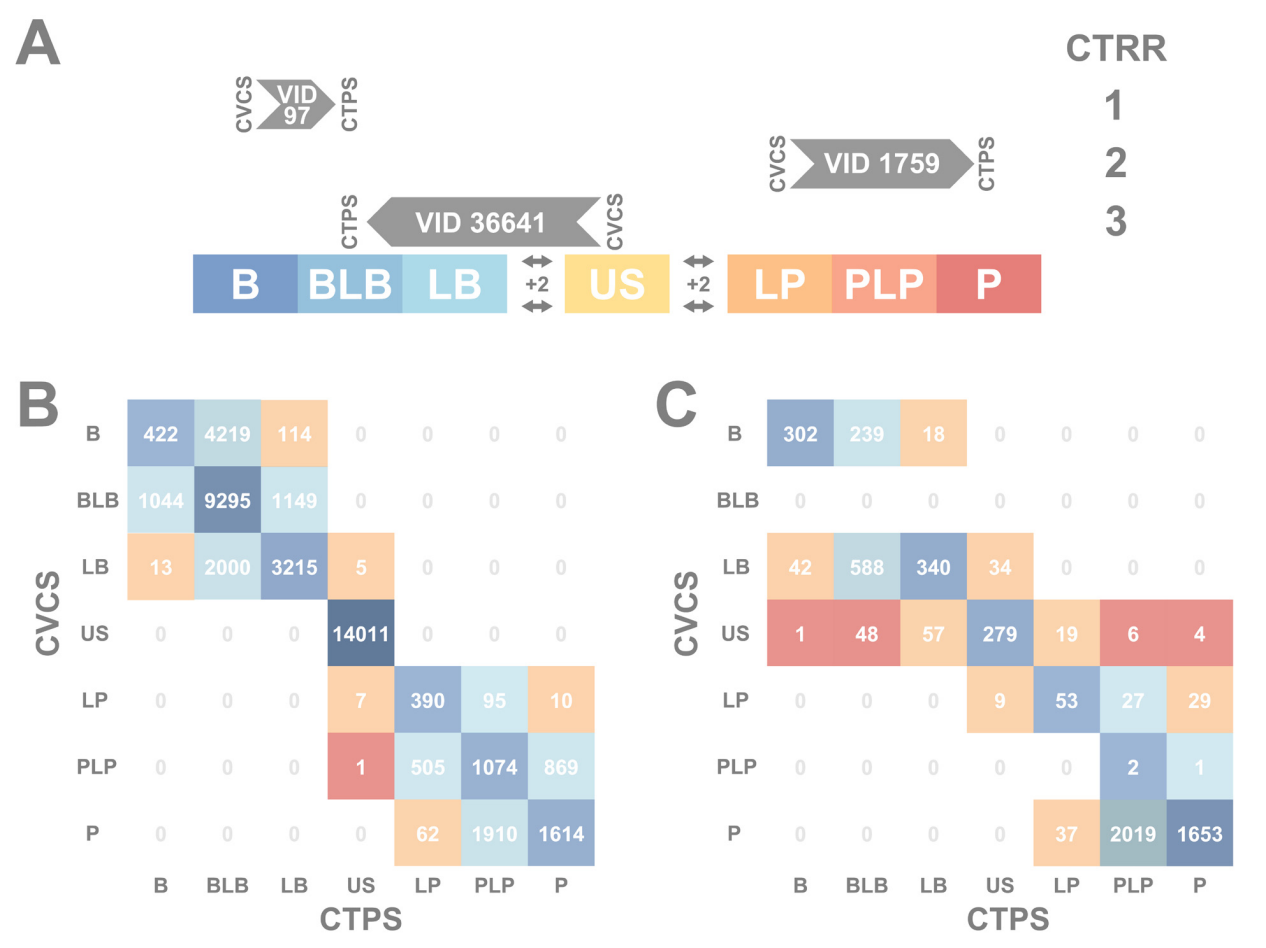
Clinotator reclassification recommendations (CTRR). ( **A**) A schematic of the CTRR scoring workflow. ClinVar Clinical Significance (CVCS) is used as a starting point, and each significance passed to arrive at the Clinotator Predicted Significance (CTPS) counts as a point. Transitioning a significance family boundary adds an extra point (moving from Uncertain Significance (US) to either Likely benign (LB) or Likely pathogenic (LP)). Benign (B), Benign/Likely benign (BLB), Pathogenic/Likely pathogenic (PLP), and Pathogenic (P). ( **B**) A heat map of variant counts for two-star variants (per ClinVar’s review status star ratings), with each CVCS and CTPS combination. Darker squares correspond to higher numbers of variants. Blue represents a CTRR of zero, light blue a CTRR of one, orange a CTRR of two and red a CTRR of three. ( **C**) A heat map of variant counts for three-star variants using the same layout as ( **B**).

**Figure 3.**
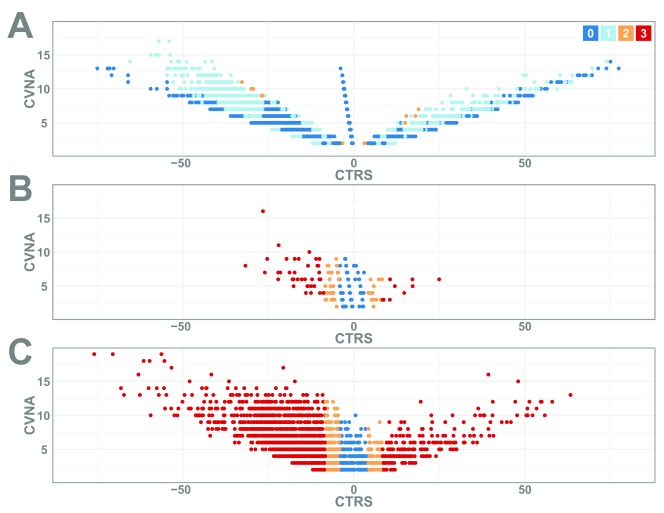
Number of clinical assertions (CVNA) given a Clinotator raw score (CTRS). ( **A**) All two-star variants plotted by CVNA and CTRS. Values are colored according to their Clinotator reclassification recommendation (CTRR): blue represents a score of zero, light blue a score of one, yellow a score of two and red a score of three. ( **B**) All three-star uncertain significance variants, plotted in the same manner and coloring scheme. ( **C**) All conflicting interpretations of pathogenicity variants.

The schematic in Figure 2A gives a demonstration of CTRR outcomes, depicted for two-star and three-star variants in Figure 2B and Figure 2C. Reclassification recommendations for all of the two-star variants in Figure 2B largely confirm that most variants shift by only a single position, if at all (see also Table 2). The most common shifts occurred between the overlap categories (BLB and PLP) and their immediate neighbors. This is likely the result of the altered definition of the overlap category, as opposed to a genuine reclassification recommendation. In Figure 3A, most two-star variants with a CTRR of zero follow one of three identifiable linear correlations between CTRS and CVNA. Given that only 212 out of 47,854 two-star variants demonstrate a CTRR more than 2, these results support previous research showing a fairly high general concordance in ClinVar ^19, 38, 39^.

Two-star variants with a CTRR of two are easily discernable in Figure 3A as four specific regions. Those with a CTRS around -27 or 15 correspond to a LB to B or LP to P transition, respectively, and each have at least five valid clinical assertions, giving them a high enough CTRS score to be good candidates for reclassification. The remaining two clusters of two-star variants with a CTRR of two represent the other four squares in Figure 2B: B to LB, P to LP, LB to US, and LP to US. All feature CTAA values ranging from 4.5 to 8 years of age, and each has exactly two valid clinical assertions. These variants may not strictly have a higher chance of reclassification, but have fairly weak and aging evidence. They would benefit most from a more recent assertion or a downgrade. The single two-star variant with a CTRR of three similarly has only two older assertions and a CTAA of 4.5 years. In this case, VID 35616 has two LP clinical assertions and an OMIM literature review from 2007 with a P, generating a PLP that in the future would be probably benefit from at least a reclassification to LP.

### ClinVar three-star variants

The 5,807 three-star variant reclassification recommendations are depicted in Figure 2C. This distribution is notably different than two-star variants; expected given an overall CVCS based on the—in most cases single—expert review assertion. There are no overlap variants in BLB, and only three in PLP. The majority of variants still have CTRR values of zero or one, but more three-star variants had a CTRR of two, 4.2% versus 0.4% of two-star variants. All but one of the high priority for reclassification variants (CTRR = 3) were in the three-star group, and these stand out noticeably in the comparison of Figure 2. All 59 of these three-star, CTRR rank three variants were of the US classification further explored in Figure 3B. This contrasts Figure 3A, as the CTRR score has no correlation with the CVNA, instead following lower bounds for CTPS ranges. This pattern is similarly observed in Figure 3C with variants of CI significance.

As Figure 3B suggests, these three-star variants with a CTRR of three are primarily predicted to be in the benign family (48 BLB, 1 B). In total, 10 are predicted to be medically significant, belonging to the pathogenic family (6 PLP, 4 P). The submitters with assertion criteria for these 10 are examined in greater detail in Table 3. In 5/10 cases, the expert assertion is the oldest, three of which are approaching 5 years of age. Additionally, there is a high level of consensus among the three most represented clinical laboratories, with at least two asserting a P or LP in 8/10. It is also notable that 8 of the variants are associated with cancer (Variation ID 42965 is associated with hypertrophic cardiomyopathy). Yang *et al.* previously found similar trends in clinical lab concordance, age-related discordance and highest concordance among hereditary cancer genes ^19^. As the expert review assertion in three-star variants overrides the other assertions, the tiered system likely disadvantages these variants, making them ideal candidates for reclassification. The full list of 60 variants with a CTRR score of three is available in Supplementary Table 1.

**Table 3.** Uncertain significance variants with three stars and a medically significant reclassification recommendation of three. VID, Variation ID; rsID, dbSNP reference SNP ID; CTPS, Clinotator predicted significance; CTRS, Clinotator raw score; CVNA, ClinVar number of clinical assertions; CTAA, average clinical assertion age; clinsig, clinical significance of assertion; LB, Likely Benign; US, Uncertain Significance; LP, Likely Pathogenic; P, Pathogenic; PI, prediction interval.

VID	rsID	CTPS	CTRS	CVNA	CTAA	date	clinsig	date	clinsig	date	clinsig	date	clinsig	date	clinsig	date	clinsig	date	clinsig
38183	81002812	P	24.903	6	1.666666667	4/15/2016	US	9/16/2016	P			8/18/2017	P	9/3/2014	LP	10/2/2015	P	3/1/2016	P
42965	397516187	PLP	11.97	5	2	12/15/2016	US			5/13/2014	P			7/22/2016	LP	9/25/2013	LP	7/24/2017	LP
89172	587779204	PLP	10.569	6	1.333333333	9/5/2013	US	8/30/2017	LP	7/7/2017	LP	5/15/2017	US	5/10/2017	US	5/17/2016	P		
90099	267607893	PLP	8.469	3	2.333333333	9/5/2013	US			7/2/2015	LP			7/5/2016	P				
91028	63751469	PLP	8.703	3	0.666666667	10/28/2015	US	6/9/2017	P	9/7/2017	LP								
91030	63750084	P	14.703	4	2.25	10/28/2015	US	5/27/2014	P	10/20/2014	P	7/14/2017	P						
91033	63750808	PLP	8.403	3	1.333333333	10/28/2015	US	8/17/2017	P	1/18/2016	LP								
91035	63751007	PLP	10.503	3	1.666666667	10/28/2015	US	6/20/2014	P			12/20/2017	P						
91328	267608161	P	17.169	5	1.4	9/5/2013	US	7/12/2017	LP	4/26/2017	P	12/5/2017	LP	3/25/2016	P				
91330	587779335	P	17.103	6	1.166666667	11/24/2015	US	11/21/2017	P			7/9/2017	P	10/30/2014	LP	7/14/2016	LB	3/27/2017	P
						**Expert reviewer**		**High volume submitters: clinical assertion, criteria provided Ambry** **Genetics (L), GeneDx (M), Invitae (R)**						**Clinical assertion, criteria provided**							

### ClinVar variants of conflicting interpretation

**Figure 4.**
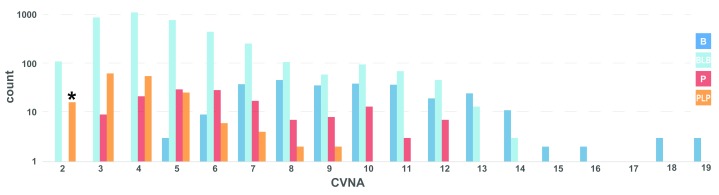
Conflicting interpretation of pathogenicity (CI) variants by number of clinical assertions (CVNA). CI variants with a Clinotator reclassification recommendation (CTRR) of three, counted by CVNA and colored by Clinotator predicted significance (CTPS). Blue represents Benign (B), light blue represents Benign/Likely benign (BLB), orange represents Pathogenic/Likely pathogenic (PLP) and red is Pathogenic (P). The asterisk denotes the column of 16 variants examined in Table 4.

One-star variants with CI status comprise a set of 13,762 variants with obvious reclassification value, as CI defines variants of all types with at least one dissenting assertion. These variants are shown in Figure 3C, and show a similar trend to the Figure 3B distribution of three-star US variants: more heavily distributed towards benign, and with CTRR distribution defined by the CTPS lower bounds. This makes sense given that CI is scored as US, so CTRS deviations should proceed from the center of the scale much like an unknown variant. Looking at the distribution of CI variants with a CTRR of three ( Figure 4), there are a number of potential reclassifications, which is unsurprising given their conflicted status. To sample what constitutes a minimum amount of evidence for a CTRR of three, the medically significant variants with only two criteria-based clinical assertions are provided in Table 4 (16 variants of PLP significance). Unlike the variants in Table 3, the majority of these variants are not associated with cancer. Instead, they are associated with cardiovascular diseases, metabolic diseases, and Rett syndrome. Despite not being cancer-focused, there is still a fair amount of concordance among clinical lab assertions. In most cases, the reason for conflict is a single significance provided without assertion criteria, substantially older than the two valid assertions. Given the ages of the conflicting assertions, and the lack of assertion criteria, inviting the submitters to re-evaluate their submissions would most likely reconcile the discrepancies. The full set of CI variants with a CTRR of three are available in Supplementary Table 2.

**Table 4.** Conflicting Interpretation variants with two criteria-driven assertions and a medically significant reclassification recommendation of three. VID, Variation ID; rsID, dbSNP reference SNP ID; CTPS, Clinotator predicted significance; CTRS, Clinotator raw score; CTAA, average clinical assertion age; clinsig, clinical significance of assertion; B, Benign; LB, Likely Benign; US, Uncertain Significance; LP, Likely Pathogenic; P, Pathogenic; PLP, Pathogenic/Likely pathogenic.

VID	rsID	CTPS	CTRS	CTAA	date	clinsig	date	clinsig	date	clinsig	date	clinsig
10768	199473684	PLP	10.8	1.5	10/7/2014	P	1/2/2018	P	4/1/2002	US	1/5/2007	P
12348	121912652	PLP	11.4	1.5	5/23/2016	P	9/23/2016	P	-	US	11/30/1990	P
18011	121909551	PLP	12	0.5	6/14/2016	P	1/19/2018	P	3/1/1992	US	6/1/2014	LB
54153	80357164	PLP	8.4	1.5	10/2/2015	P	10/17/2016	LP	1/31/2014	US		
55564	80358073	PLP	10.8	2	10/2/2015	P	9/21/2015	P	12/7/2011	P	8/26/1998	US
93457	61748906	PLP	9.6	2.5	12/19/2012	P	6/30/2017	P	4/30/2017	LP	-	B
143603	61748420	PLP	9.6	2.5	2/8/2013	P	6/13/2017	P	2/15/2011	US		
143738	61749723	PLP	8.7	1.5	11/16/2016	P	6/30/2015	LP	1/21/2008	US		
156661	587783132	PLP	11.4	1.5	6/15/2016	P	9/11/2015	P	11/5/2009	US		
161516 ^*^	193920774	PLP	9	1	10/7/2016	LP	8/3/2016	P				
185705	80356913	PLP	8.4	1	6/26/2017	LP	10/2/2015	P	1/31/2014	US		
201215	794728721	PLP	12	1	10/10/2016	P	5/3/2017	P	3/19/2015	LP	-	US
202509	768431507	PLP	9	0.5	8/7/2017	P	2/15/2017	LP	3/26/2014	US		
203805	150591260	PLP	12	1	4/24/2017	P	5/5/2017	P	2/29/2016	P	10/31/2017	US
205867	796052621	PLP	11.4	1.5	10/28/2015	P	6/13/2016	P	9/30/2016	US		
280584	886041761	PLP	8.4	1.5	5/18/2016	P	2/14/2017	LP	12/31/2016	US		
					**Clinical assertion, criteria provided**				**Clinical assertion, no criteria provided OR literature review**			

[i] * variant has somatic variant interpretations, 18 LP and 1 US

Notably, one of the variants in this list, VID 161516, had a CI significance based on one P, one LP, 18 LP (somatic) and one US (somatic) assertions. The literature has largely not addressed how to reconcile somatic and germline assertions, and the ACMG/AMP guidelines explicitly state they are “not intended for the interpretation of somatic variation” ^4^. ClinVar appears to take somatic mutation into account on a limited scale, but as more somatic data is submitted to ClinVar this may need to be addressed by the ACMG/AMP recommendations, or excluded from overall significance estimation.

## Discussion

### Usefulness of Clinotator

As shown above, Clinotator is a useful secondary analysis tool for identifying discrepant records amongst the large and complex ClinVar database. With limited resources, submitters and curators alike can utilize Clinotator metrics for prioritization of reclassifications and research. Additionally, Clinotator can be used to obtain ClinVar information in batch annotations, providing a convenient method to rapidly obtain some simple ClinVar metrics and Clinotator metrics with minimal computational effort. It can be readily integrated into existing pipelines or stand alone as a quick reference.

Clinotator’s ability to identify and filter missing data fields can also be leveraged to clean up older or incomplete submissions in ClinVar. For instance, the list of variations with at least two stars returned over 9000 assertions with a blank ‘Date Last Evaluated’ field, which has become a required field for current submissions. Submitters can check their own assertions to identify their submissions that lack an assertion date.

It should also be noted that Clinotator should not be used as a tool for directly determining clinical significance. Although Clinotator does develop a predicted significance, this is not through the use of primary evidence. The predictive range generated is for rating evidence strength and reclassification impact. Reclassification should always be done using the ACMG/AMP guidelines and assessing all primary evidence available to the researcher.

### Aggregate scoring rationale

To compare/analyze variation report quality (a secondary analysis), Clinotator attempts to establish some common criteria. How to combine independent analyses is a particular problem, as these are not individual data points, but professional judgements using a coordinated guideline and overlapping evidence. It has been previously noted that there will always be some level of professional judgement that results in incongruous assertions ^11^, but ultimately this needs to be reconciled to arrive at an overall interpretation by consensus. Mean or median assertion values will not account for the total body of assertions, falling prey to skew or omission, respectively. This is particularly so when there are multiple weighting factors modulating assertion values, thus an aggregated score can better express the total volume of assertions. Clinotator utilizes its raw score, which is an aggregate of these weighted clinical assertions.

A potential issue that arises out of an aggregate model is that lower-level assertions made in a larger volume might artificially inflate the overall value of a variant. For instance, five LP assertions may give a variant the P status, despite no one submitter having enough evidence for the P category. However, while individual assertions utilize overlapping data, each one likely possesses additional private data as evidence. Thus each LP assertion does provide an additive value in terms of overall pathogenicity. We should therefore consider the five ‘Likely pathogenic’ assertions as more likely ‘Pathogenic’. Clinotator highlighted the two-star variants falling in this hypothetical category as a prime candidates for data sharing and reconciliation between the submitters to reach concordance. Clinotator is calibrated on the current, unambiguous two-star data in ClinVar and will be recalibrated on a regular basis to ensure that these boundaries: (i) change with richer information being submitted to ClinVar, and (ii) honor the intent of the ClinVar starring system when possible. In the ideal case, all of the submitters to ClinVar would have all of the data available and the resources to analyze all variants in ClinVar on a regular basis. In such an optimistic context, Clinotator would likely consider a mean/median model.

### Assertion weight and prediction intervals

Assigning assertion weights to significance types is unfortunately a subjective process. There is not a universal, objective measure of quantity of pathogenicity available in ClinVar, or, arguably, in the literature. In lieu of a more objective metric, a range of assertion weights were tested and the control two-star distributions were examined, as was the set of all two-star variants (42,717 variants; Figure 1). This allowed for the analysis of variants with mixed assertion types, including analysis of CI variants and variants with mixed submitter expertise categories. For the assertion weights we tried, the relative shapes and overlaps of the five control distributions were largely consistent with the final values. Larger assertion weights primarily expanded the tail skew and overall CTRS values, while smaller weights lowered the distance between US and the other classes, shrinking all CTRS values. Expanding the distance between “Likely” and full class members similarly modified overall overlap CTRS values, but the relative overlap trend (that BLB carried a wider range than PLP) did not change. Mixed assertions can never be separated. Ultimately, Clinotator’s assertion weights are relative to class control distributions, so the current values were spread enough to observe comparative differences in overlap, while delineating pathogenicity families with a high degree of confidence. Future versions of Clinotator will need to be periodically recalibrated on current ClinVar distributions, and ultimately may weight assertion types differently if a more objective standard becomes available.

The PI ranges themselves are defined more objectively. As the control distributions are non-normal in several respects, a ranked non-parametric PI is most appropriate ^27^, relying on the fairly large cross sections of the total variant sets in ClinVar ( Table 2). Simply setting a static confidence level for the PI would be preferred, but as the lower bound is set by the ClinVar two-star criterion, scaling the whole range is far better than modifying it. As a result, there is a higher confidence in the predictions of some classes than others, but all are at least 95% confident ( Figure 1B). As the goal of these prediction ranges is to assess evidential disparities and not to definitively classify variants, having conservatively wide ranges ensures a higher specificity for the CTRR statistic.

### Submission age and submitter expertise

The age of the assertion matters. This has been previously identified as an issue ^6, 11, 19^, but as both the test cases demonstrate, outdated assertions often fail to take into account new evidence and negatively impact classification. One of the key benefits of the current ACMG/AMP criteria is that any assertion must review all previous evidence and existing data available ^4^. Note that this should not include old assertions, only evidence. Thus while old data never loses its value, old assertions do; particularly if they were made prior to the establishment of the current standards. Reclassification on a regular interval should be a goal for submitters to ClinVar. Counter to the concept of clinical significance as a static value, it is intrinsically dynamic based on the limited availability of evidence. Thus Clinotator weights against the age of assertions after a grace period to ensure that current literature and data are more effectively integrated into the variation report. The maximum threshold of the age weight was set to 0.5 so that a P or B (±6) assertion at or beyond the limit is effectively downgraded to the associated “Likely” category (±3). LB or LP assertions cannot be downgraded to US significance, but are similarly halved in strength.

Finally, the submitter expertise category is a continued confounder in ClinVar. It has become essential for experts in individual conditions to become involved in classification, as different conditions have nuanced profiles of pathogenicity ^7, 11^. However, as seen in our test cases, having the expert reviewers supersede all other clinical assertions results in a masking of assertion data. This complication is exacerbated by the age of assertion issue, but more frequent reclassification wouldn’t address the tiered nature of system. Clinotator’s solution is to weight by reviewer status, giving expert reviews a louder voice without drowning out the clinical significance conversation. The specific weights for expertise are subjective, owing to the absence of an objective submission quality metric on which to rank submitter expertise.

### Phenotypic classification

Some phenotype information is reported by Clinotator: the conditions associated with the submitter’s assertion. While it is possible to split assertions by condition and develop a clinical significance for each, this is currently too problematic to implement. For example, VID 9 has entries associated with “Hereditary hemochromatosis,” “Hemochromatosis type 1,” “Hemochromatosis type 1 (Autosomal recessive inheritance),” “Hemochromatosis juvenile digenic,” and “not provided” all with varying or absent identifiers in phenotype databases (MedGen, Human Phenotype Ontology, OMIM). As these are all potentially ambiguous classifications of the same or similar conditions, it would difficult to effectively group them without more comprehensive standardization.

Additionally, some submitters provide a single assertion with multiple conditions associated, while others provide multiple assertions per variant, one for each condition. And some variants have two assertions total (as with VID 267572), which differently describe the same condition (hereditary breast and ovarian cancer). If these were split, there would not be enough information for Clinotator to calculate metrics on either. Of the variants examined here, 19,249 have only two valid clinical assertions with differing conditions for each; potentially excluding almost a third of ClinVar variants with multiple assertions. As ClinVar evidence grows, and phenotype ontologies become more sophisticated, it will become more feasible to split variant assertions by phenotype.

### Future directions

Next steps for development involve a variety of fine tuning work. As its metrics are used for analysis, their effectiveness can be assessed and modified, particularly those with subjective elements. An ideal scenario for assertion type weighting would be for submitters to declare the evidence types they utilized, and whether that came from a private resource (i.e. PS4, private data; or PM2, ExAC data). This would allow for assertion type scoring based on an aggregate of evidence without overlap.

Similarly, as variant annotations are tracked over time, the submitter expertise category can be calibrated to reflect the total body of experience that a submitter has, or the relative rates of reclassification in the different review status tiers. With more longitudinal data on variants as ClinVar grows, it may become possible to establish a submitter expertise structure based on number of assertions submitted, relative reassessment rate and/or number of misclassified variants.

The above examples only begin to describe Clinotator’s applications. Clinotator presents a framework for quantitatively assessing ClinVar evidence, and exploration of variants that have unusual Clinotator metrics. Clinotator can also incorporate new utilities to improve its data parsing sophistication, and additional metrics can be included, potentially incorporating new factors such as somatic mutation. Hopefully, it will become a useful tool for curation of ClinVar, and can be integrated with other tools, allowing for the improved classification of variants.

## Data and software availability

RRID: SCR_016054.

Clinotator source code available from: https://github.com/rbutleriii/Clinotator.

Archived source code at the time of publication: https://doi.org/10.5281/zenodo.1210204 ^40^.

Software license: GNU General Public License v3

Raw data and analysis is available at: https://doi.org/10.5281/zenodo.1285151 ^41^.

## Supplementary material

**Supplementary Table 1. ClinVar three-star variants of high priority for reclassification.**

A table of the output annotations for ClinVar three-star variants with a Clinotator reclassification recommendation score of 3.

**Supplementary Table 2. ClinVar conflicting interpretation variants of high priority for reclassification.**

A table of the output annotations for ClinVar ‘conflicting interpretation of pathogenicity’ variants with a Clinotator reclassification recommendation score of 3.
