# Supplementary material for: Clinotator: analyzing ClinVar variation reports to prioritize reclassification efforts
Source: F1000Res. 2018 Jun 20;7:462. Originally published 2018 Apr 13. [Version 2] doi: 10.12688/f1000research.14470.2 (PMC5941247; doi:10.12688/f1000research.14470.2)
Supplement: Peer review history

# Peer review: Referee response for version 1

**DOI:** 10.5256/f1000research.15752.r33596

**Published:** 2018-05-08

**Authors:** Amalio Telenti

**Affiliations:** 1 Scripps Translational Science Institute, Scripps Research Institute, La Jolla, CA, USA

Genetic testing is increasingly considered in clinical grounds, and may accelerate as payers approve specific applications. The clinical validity is built on substantial evidence for pathogenicity of individual variants, in particular those that have been vetted by expert panels, and exceptionally, endorsed by guidelines. This parsimonious implementation of genetic information in the clinics (evidence, expert support, guidelines and reimbursement) is difficult because current techniques provide large amount of information that is incompletely assessed. Thus, only a small proportion of known variants are confidently classified and the community and corporate efforts aim at creating standards at the same time that a legacy of genetic variant interpretation is being updated. Prime among the efforts is ClinVar, a public database with information on over 300,000 variants. ClinVar uses various approaches to classify variants on the basis of the quality of evidence; and by doing so, exposes the number of entries that are conflicting, or just of unknown significance. ClinVar creates a platform that supports the ongoing classification and correction of errors. 

 Clinotator, described in this present publication, aims at highlighting entries that may need reappraisal. It does it primarily for batch analysis of variants or genes. This is achieved by building weights – mostly through heuristics – around key factors that contribute to misclassification: the age of the submission and last review of evidence, the standing of the submitter. The program results in a metric of pathogenicity and an alert for the need of reclassification. Calibration of the metrics and weights is achieved by using subsets of ClinVar variants with established criteria.

 Clinotator adds to other efforts to improve the quality of annotation, most notably ClinVar Miner ( https://clinvarminer.genetics.utah.edu). However it is important to underscore that other criteria, beyond the age of record, review of evidence and submitter, are important in the overall effort – in particular the use of disease prevalence ^1^, and the consideration for penetrance of variants ^2^. Given the current emphasis on machine learning in genetics and genomics, it is conceivable that a more comprehensive modeling of evidence and of biological basis of deleteriousness (eg, pathogenicity scores such as CADD ^3^) may contribute increasingly accurate ascertainment of variants.

 Given the considerations above, there are two aspects of the work that merit attention: (i) a more complete representation of the contribution of Clinotator in the context of other efforts, and (ii) a discussion of the likelihood that the current algorithm simply reclassifies variants between the overlap categories and their immediate neighbors. It would be useful to evaluate the accuracy of the reclassification by an external expert panel.

I have read this submission. I believe that I have an appropriate level of expertise to confirm that it is of an acceptable scientific standard.

## References

[1] ShahNHouYCYuHCSaingerRCaskeyCTVenterJCTelentiA: Identification of Misclassified ClinVar Variants via Disease Population Prevalence. *Am J Hum Genet*.2018;102(4) : 10.1016/j.ajhg.2018.02.019 609-619 10.1016/j.ajhg.2018.02.019 29625023PMC5985337

[2] YangSLincolnSEKobayashiYNykampKNussbaumRLTopperS: Sources of discordance among germ-line variant classifications in ClinVar. *Genet Med*.2017;19(10) : 10.1038/gim.2017.60 1118-1126 10.1038/gim.2017.60 28569743PMC5632819

[3] KircherMWittenDMJainPO'RoakBJCooperGMShendureJ: A general framework for estimating the relative pathogenicity of human genetic variants. *Nat Genet*.2014;46(3) : 10.1038/ng.2892 310-5 10.1038/ng.2892 24487276PMC3992975

# Response

**Published:** 2018-06-08

**Authors:** Robert Butler

**Affiliations:** NorthShore University HealthSystem, USA

Thank you very much for the review, and the perceptive considerations posed. I reworked the analysis and paper to highlight the topics suggested by the reviewer and include the recommended references. Below are specific considerations:

                

*(i) a more complete representation of the contribution of Clinotator in the context of other efforts*

 I reworked several elements to better define the context for Clinotator. Particularly in the Introduction and Discussion, I spell out the role of Clinotator as a secondary analysis tool for comparing primary analyses: individual clinical assertions.

 “Harrison *et al.* found that 87.2% of discordant variants were resolved by reassessment and data sharing ^8^ . New public data has recently been leveraged with private datasets to identify misclassified variants on the basis of variant penetrance given disease prevalence [ins cit] . However the majority of these reclassification efforts still rely on access to private data, which will continue to be an unavailable to most researchers for the foreseeable future.”

 “Since it is based entirely on data available in ClinVar, it requires no private dataset or access to external resources. To demonstrate its utility, we examined test sets of two-star, three-star, and four-star variants (per ClinVar’s review status star ratings) and variants in ClinVar with “Conflicting Interpretations of pathogenicity” (CI). Clinotator was able to confirm recently published concordance trends ^6^^, ^^8^^, ^^18^ , and identify several groups of discordant variants for further investigation. It accomplished this efficiently, using a large-scale systematic approach with a minimal computational effort.”

 “As shown above, Clinotator is a useful secondary analysis tool for identifying discrepant records amongst the large and complex ClinVar database. With limited resources, submitters and curators alike can utilize Clinotator metrics for prioritization of reclassifications and research.”

 Part of the usefulness of Clinotator as a secondary analysis is its lightweight nature, not requiring the user to have their own cohort data. If we modify Clinotator to include primary evidence in its analysis—including penetrance, prevalence, and prediction software—that runs the risk of a circular set of self-reinforcing assertions, built on other assertions which may already include those evidence categories.

 Still, those primary evidence types above are very valuable, and should be factored into new clinical significance assertions (with criteria provided). The evidential papers themselves can be entered into ClinVar as “Literature Reviews” evidence type for associated variants. Ideally then, that information can be readily incorporated into all of the subsequent assertions as they become more frequently updated. Tools like those mentioned in the introduction (InterVar, ClinVar Miner, MyVariant.info, HGMD, SCRP, etc.) and various annotation software are then the appropriate resources for generating updated primary assertions.

*(ii) a discussion of the likelihood that the current algorithm simply reclassifies variants between the overlap categories and their immediate neighbors.*

 The reviewer is correct in noticing that the majority of CTRR=1 variants are likely shuffling between overlap categories and their neighbors. To make this stand out better in the paper, I expanded the analysis from the two use case scenarios (3-star uncertain and Conflicting Interpretation) to an overall assessment of variant classifications in two-star, three-star, four-star and conflict categories.  Updating figures 2, 3, the Results and Discussion to this end (with software modifications suggested by Reviewer 1, the entire analysis was redone). Some relevant modifications:

 “For this purpose, the overlapping regions of PLP and BLB exist—not as yet another classification bin—as a measure of plasticity of borderline assertions. The quantitative nature of the CTRS also allows a given variant to transition out of the overlap should enough additional assertions arise, or if sparse limited assertions are not updated.”

 “The schematic in Figure 2A gives a demonstration of CTRR outcomes, depicted for two-star and three-star variants in Figure 2B and Figure 2C. Reclassification recommendations for all of the two-star variants in Figure 2B largely confirm that most variants shift by only a single position, if at all (see also Table 2). The most common shifts occurred between the overlap categories (BLB and PLP) and their immediate neighbors. This is likely the result of the altered definition of the overlap category, as opposed to a genuine reclassification recommendation. In Figure 3A, most two-star variants with a CTRR of zero follow one of three identifiable linear correlations between CTRS and CVNA. Given that only 212 out of 47,854 two-star variants demonstrate a CTRR more than 2, these results support previous research showing a fairly high general concordance in ClinVar ^18^^, ^^37^^, ^^38^ .”

 “The 5,807 three-star variant reclassification recommendations are depicted in Figure 2C. This distribution is notably different than two-star variants; expected given an overall CVCS based on the—in most cases single—expert review assertion. There are no overlap variants in BLB, and only three in PLP. The majority of variants still have CTRR values of zero or one, but more three-star variants had a CTRR of two, 4.2% versus 0.4% of two-star variants. All but one of the high priority for reclassification variants (CTRR = 3) were in the three-star group, and these stand out noticeably in the comparison of Figure 2.”

 These are some of the modifications regarding the overlapping categories, in a reworked analysis. This also makes the direction of the analysis clearer, and allowed me to flesh out discussion of the overlap categories and the benefit of redefining overlaps with a quantitative scale. I also then highlighted that conflicting interpretations and older 3-star assertions are key focal points for Clinotator.

*It would be useful to evaluate the accuracy of the reclassification by an external expert panel.*

 Any submitter that properly conforms to the ACMG/AMP guidelines for an assertion is theoretically giving an expert opinion, but as Harrison, *et al* (2017) point out, there will always be disagreement as long as some level of professional judgement is involved in the determination. Thus, in the absence of an absolute correct standard, a consensus of professional judgements is the aim for Clinotator. I further detailed the Future Directions section to describe our next steps in terms of developing a longitudinal model for submitter accuracy in terms of historical classification:

 “…or the relative rates of reclassification in the different review status tiers. With more longitudinal data on variants as ClinVar grows, it may become possible to establish a submitter expertise structure based on number of assertions submitted, relative reassessment rate and/or number of misclassified variants.”

# Peer review: Referee response for version 1

**DOI:** 10.5256/f1000research.15752.r33507

**Published:** 2018-05-02

**Authors:** Kai Wang, Gangcai Xie

**Affiliations:** 1 Children's Hospital of Philadelphia, Philadelphia, PA, USA

In this study, the authors introduced a new method named Clinotator to help researchers easily find the discrepancies on categorizations of clinical impacts among different submitters or curators. Besides providing re-classification prioritization scores, this study also provided new metrics (CTRS, CTAA, CTPS, and CTRR) for the interpretation ClinVar variants, which will be helpful for users of ClinVar data. Some additional improvements on description of the methods and interpretation of results can be made as described below.

 Major:

1. A brief description about the selection of the values for the assertion weight factor can be added to the methods. Although there is a discussion about this in the discussion part, it is not present in the Methods itself.
2. Based on Figure 2A, it is still unclear how the CTRR (Clinotator reclassification recommendations) system works. The legend says “scoring workflow”, but this is not really a workflow figure. Maybe a flowchart with a simple example will help (for example, to show how moving from Uncertain Significance (US) to Benign/Likely benign (BLB) scores works for a particular variant based on a specific reason).
3. For the definition of CTRR in page 4, is it suitable to define both the cases with “insufficient information” and “consistent identity” to be 0? It seems that the cases with “insufficient information” have a good chance to be re-classified in the future when more information is available.
4. In the discussion part, the authors mentioned that Clinotator might classify the variant with five LP assertions as P status, and this issue was further explained by the authors through the fact that those LP assertions do provide additive values for overall pathogenicity. However, in the further discussion, the authors mentioned that “One of the key benefits of the current ACMG/AMP criteria is that any assertion must review all previous evidence and existing data available”, which means that the latest assertions were dependent on the previous ones. Will this affect the additive assumption? Maybe a discussion about this can help.
5. rsID does not uniquely identify a variant. It is merely a locus identifier telling people an approximate genomic location. In general it is a bad idea to use rsID to denote a variant, especially since many variants can be at the exactly same locus (3 different SNPs, and many different indels can all be located at exactly the same genomic position and be represented by exactly the same rsID). ClinVar does assign its own ID, and it is a better idea to just use ClinVar’s own ID system when describing variants.
6. One important discussion point is the use of phenotype information in the clinical interpretation of genetic variants. It can be incorporated in ACMG guidelines, but here for ClinVar, there is no phenotype information associated with a variant ID, so a pathogenic variant for one phenotype could simply be benign for another phenotype. It is something that needs to be discussed.

Minor:

1. The word “Benign ( **B**)” is in bold font in both of Figure 1 and Figure 2 legend. This is very confusing, because there is also an explanation of the panel (A), (B), etc.
2. Too many abbreviations were used in the manuscript. It would be ideal to make a list to explain each of them? For example, in Table 3, there is no further explanation for VID, clinsig, even though there are explanations for other abbreviations.
3. Both “rsID” and “RSID” are used in the manuscript, a more consistent naming scheme is needed.
4. How is the “age of assertion factor” defined? Is it the current date (i.e. the date of preparing the manuscript, Feb 2018) minus the date of assertion? Maybe a clear description about this will help readers get a better understanding.
5. In Table 3, it is better to use “Ambry Genetics” rather than “Ambry”
6. In the “Data and software availability” part, should the Clinotator source code be https://github.com/rbutleriii/Clinotator rather than “ https://github.com/rbuteriii/clinotator”?

We have read this submission. We believe that we have an appropriate level of expertise to confirm that it is of an acceptable scientific standard.

# Response

**Published:** 2018-06-08

**Authors:** Robert Butler

**Affiliations:** NorthShore University HealthSystem, USA

Thank you very much for the review. It was a very helpful and I went through and made changes to the manuscript and program, reflected below. The result is a new version of Clinotator, and a rerun of the original variant set, with updated analysis and figures to reflect the changes.

**Major**

*1.   A brief description about the selection of the values for the assertion weight factor can be added to the methods. Although there is a discussion about this in the discussion part, it is not present in the Methods itself.*

 I added an explanation of assertion weights to the methods, referencing the later Discussion section as well:

 “Initial values of US were tried from -0.5 to -0.1 in increments of 0.1, ultimately defined at -0.3. The assertion weight factor for LB was tried as several multiples of US (4, 5, 6, 10, 12, and 20). A 10-fold multiple, with LB equal to -3, eliminated overlap between US and LB distributions. The value of B was tested at a range of multiples of LB (3/2, 5/3, 2, 3, and 4), and fixed at a 2-fold value, with a B assertion weight of -6. No multiple of US could attain LP, which was set as the equivalent positive value to LB; and P was set 2-fold higher than LP (after trying a range of multiples as with B). Further rationale of weights is continued in the Results and Discussion sections.”

*2.   Based on Figure 2A, it is still unclear how the CTRR (Clinotator reclassification recommendations) system works. The legend says “scoring workflow”, but this is not really a workflow figure. Maybe a flowchart with a simple example will help (for example, to show how moving from Uncertain Significance (US) to Benign/Likely benign (BLB) scores works for a particular variant based on a specific reason).*

 Figure 2 was reworked to better depict the CTRR classification and reclassifications by CVSZ. See figure.

*3.   For the definition of CTRR in page 4, is it suitable to define both the cases with “insufficient information” and “consistent identity” to be 0? It seems that the cases with “insufficient information” have a good chance to be re-classified in the future when more information is available.*

 The reviewer makes an excellent point, I modified the software to separate CTRR=0 as consistent identity only. Insufficient information variants now receive a CTRR= “.”. Version 1.1.0 onward should reflect this change.

*4.   In the discussion part, the authors mentioned that Clinotator might classify the variant with five LP assertions as P status, and this issue was further explained by the authors through the fact that those LP assertions do provide additive values for overall pathogenicity. However, in the further discussion, the authors mentioned that “One of the key benefits of the current ACMG/AMP criteria is that any assertion must review all previous evidence and existing data available”, which means that the latest assertions were dependent on the previous ones. Will this affect the additive assumption? Maybe a discussion about this can help.* 

 I rephrased the section of the discussion to make this clear.

 “One of the key benefits of the current ACMG/AMP criteria is that any assertion must review all previous evidence and existing data available ^4^ . Note that this should not include old assertions, only evidence. Thus while old data never loses its value, old assertions do; particularly if they were made prior to the establishment of the current standards.”

 Additionally, in the Methods section:

 “Note that the CTRS metric only includes clinical assertions where the submitter has published a defined assertion criteria on the ClinVar website. Literature-only submissions such as those from OMIM are filtered out as they are a type of evidence, and not a clinical assertion. Assertions made without assertion criteria or with incomplete data are also omitted, as the reliability of these assertions is unknown.”

*5.   rsID does not uniquely identify a variant. It is merely a locus identifier telling people an approximate genomic location. In general it is a bad idea to use rsID to denote a variant, especially since many variants can be at the exactly same locus (3 different SNPs, and many different indels can all be located at exactly the same genomic position and be represented by exactly the same rsID). ClinVar does assign its own ID, and it is a better idea to just use ClinVar’s own ID system when describing variants.* 

 A fair consideration about rsIDs. In the Operation section I added a paragraph on data types to clarify the related concerns:

 “The preferred input file types are a VID list or a vcf file. The rsID list alone is inherently ambiguous, as multi-allelic rsIDs can have several VIDs associated, one with each allele. The rsID to VID conversion is not 1:1, so the table file generated will return rows for all possible VIDs associated with the rsID. Thus an rsID generated table may require additional matching using the alternate allele column (CVAL). However, vcf files will only be annotated with the correct rsID/alternate allele combination, preventing a mix-up for the vcf input type. Conversely some VIDs have multiple rsIDs, either because they are a haplotype variant, or due to other complications with rsID curation. The ‘vcf_match’ field addresses this reverse situation by identifying all rsIDs associated with a VID and its haplotype allele status.”

 I also added the alternate allele “CVAL” field description (formerly CVMA) to Table 1. It was already in the Clinotator output, and used in the backend of the vcf module, but overlooked in the paper methods.

*6.   One important discussion point is the use of phenotype information in the clinical interpretation of genetic variants. It can be incorporated in ACMG guidelines, but here for ClinVar, there is no phenotype information associated with a variant ID, so a pathogenic variant for one phenotype could simply be benign for another phenotype. It is something that needs to be discussed.*

 A section has been added to the Discussion talk about this:

 “Some phenotype information is reported by Clinotator: the conditions associated with the submitter’s assertion. While it is possible to split assertions by condition and develop a clinical significance for each, this is currently too problematic to implement. For example, VID 9 has entries associated with “Hereditary hemochromatosis,” “Hemochromatosis type 1,” “Hemochromatosis type 1 (Autosomal recessive inheritance),” “Hemochromatosis juvenile digenic,” and “not provided” all with varying or absent identifiers in phenotype databases (MedGen, Human Phenotype Ontology, OMIM). As these are all potentially ambiguous classifications of the same or similar conditions, it would difficult to effectively group them without more comprehensive standardization."

 "Additionally, some submitters provide a single assertion with multiple conditions associated, while others provide multiple assertions per variant, one for each condition. And some variants have two assertions total (as with VID 267572), which differently describe the same condition (hereditary breast and ovarian cancer). If these were split, there would not be enough information for Clinotator to calculate metrics on either. Of the variants examined here, 19,249 have only two valid clinical assertions with differing conditions for each; potentially excluding almost a third of ClinVar variants with multiple assertions. As ClinVar evidence grows, and phenotype ontologies become more sophisticated, it will become more feasible to split variant assertions by phenotype.”

 Additionally, the CVDS reporting has been modified to reflect the clinical significance associated with each condition assertion. This will hopefully integrate disease specific assertions downstream and in future versions.

**Minor**

*1.   The word “Benign (B)” is in bold font in both of Figure 1 and Figure 2 legend. This is very confusing, because there is also an explanation of the panel (A), (B), etc.* 

 Fixed the bolding in the figure legend.

*2.   Too many abbreviations were used in the manuscript. It would be ideal to make a list to explain each of them? For example, in Table 3, there is no further explanation for VID, clinsig, even though there are explanations for other abbreviations.* 

 The VID is defined in the text body, but I also added it to Table 1 with rsID for more clarity. I additionally added both to all of the appropriate online table legends, as well as the “clinsig” definition.

*3.   Both “rsID” and “RSID” are used in the manuscript, a more consistent naming scheme is needed.* 

 Converted all the instances of RSID to the more consistent rsID.

*4.   How is the “age of assertion factor” defined? Is it the current date (i.e. the date of preparing the manuscript, Feb 2018) minus the date of assertion? Maybe a clear description about this will help readers get a better understanding.* 

 Description of age calculation added to the Metric Calculations section:

 “The Clinotator average assertion age (CTAA) is the mean age (in years) of valid clinical assertions. Each assertion’s age is calculated at the time of Clinotator script execution as the number of years since the clinical significance last evaluation date. Assertions without a last evaluation date are omitted.”

 I also added a “run on” date to the terminal/logfile, and the annotated vcf has a metadata field describing the Clinotator version and date.

*5.   In Table 3, it is better to use “Ambry Genetics” rather than “Ambry”* 

 Company name corrected.

*6.   In the “Data and software availability” part, should the Clinotator source code be https://github.com/rbutleriii/Clinotator rather than “https://github.com/rbuteriii/clinotator”?* 

 Url typos corrected.
